# Hypertension attenuates the link of osteoprotegerin to reduced baroreflex sensitivity in type 2 diabetes mellitus patients on oral antidiabetic and antihypertensive therapy – a cross sectional study

**DOI:** 10.1186/s12902-022-01137-w

**Published:** 2022-09-09

**Authors:** A. Naga Sailaja, Nivedita Nanda, B. S. Suryanarayana, G. K. Pal

**Affiliations:** 1 Department of Biochemistry, Jawaharlal Institute of Postgraduate Medical Education and Research (JIPMER), Puducherry, 605 006 India; 2 Department of Medicine, Jawaharlal Institute of Postgraduate Medical Education and Research (JIPMER), Puducherry, India; 3 Department of Physiology, Jawaharlal Institute of Postgraduate Medical Education and Research (JIPMER), Puducherry, India

**Keywords:** Type 2 diabetes, Hypertension, CV risk, Baroreflex sensitivity, TNF- α, Osteoprotegerin

## Abstract

**Purpose:**

Decreased baroreflex sensitivity (BRS) has been shown to be a marker of cardiovascular (CV) risk. In the present study, the difference in CV risk biomarkers in type 2 diabetes (T2D) patients receiving oral antidiabetic drugs (OAD) with and without hypertension has been assessed.

**Materials and methods:**

Ninety-two T2D patients on OAD without hypertension (control group) and eighty-eight diabetic patients with hypertension on OAD and antihypertensive drugs (test group) matched for age, gender, body mass index, serum glucose, glycated haemoglobin, and duration of the disease were recruited for the study. Their blood pressure (BP) variability including BRS, heart rate variability (HRV), insulin, lipid profile, osteoprotegerin (OPG), and tumor necrosis factor-α (TNF-α) were estimated. The association of various factors with BRS was assessed by Spearman correlation and multiple regression analysis.

**Results:**

BRS was decreased (13.90 ± 5.27 vs 6.76 ± 4.58), HRV sympathetic indices [LFnu, LF-HF ratio (1.30 ± 0.49 vs 1.93 ± 0.62)], HOMA-IR, atherogenic index of plasma (AIP), OPG (223.08 ± 103.86 vs 287.60 ± 121.36) and TNF-α were increased, and parasympathetic indices [TP (1012.90 ± 316.18 vs 625.88 ± 229.84), RMSSD, SDNN, NN50, pNN50] were decreased in the test group compared to control group. In control group, parasympathetic indices, AIP, OPG, and TNF-α had a significant correlation and OPG had an independent association (β − 0.344; p 0.004) with BRS. In test group, BP, LF-HF ratio, parasympathetic indices, AIP, OPG, and TNF-α had significant correlation, and TNF-α alone (β − 0.297; p 0.022) had an independent contribution to decreased BRS.

**Conclusion:**

Despite antidiabetic and antihypertensive treatments, T2D patients with hypertension had more cardiometabolic risks in comparison to normotensive T2D patients. Inflammation could be the inciting factor for rise in BP and decrease in BRS (CV risk) in hypertensive T2D patients. Hypertension in diabetes could attenuate the link of OPG to the reduction in BRS. Reduction in BRS could be a physiological marker of CV risk in T2D patients treated with OAD.

## Introduction

Hypertension is present in more than 50% of diabetes mellitus (DM) patients contributing significantly to microvascular and macrovascular complications [1, 2]. Indeed, the risk for cardiovascular disease (CVD) is four times higher in patients with both DM and hypertension compared to the normotensive, nondiabetic controls [3]. The prevalence of hypertension among DM patients depends on age, gender, body mass index (BMI), ethnicity, duration of DM, etc. [4]. As hypertension is a high-risk factor for vascular complications in chronic diabetes, individuals with both DM and hypertension are at a greater cardiovascular (CV) risk than DM alone [5]. As about 75% of CVD in diabetes can be attributed to hypertension, a more aggressive treatment of hypertension in addition to glycemic control is part of the management protocol of DM [6]. Despite adequate antidiabetic treatment, a group of patients develops hypertension after a few years of suffering from the disease, whereas others do not. Therefore, it is imperative to identify the plausible mechanistic processes involved in the development of hypertension in diabetes. If these processes can be detected early, preventive measures could be adopted to halt them.

For long, the first line of drugs of choice for the management of type 2 diabetes mellitus (T2D) is sulfonylurea and biguanides, either as monotherapy or in combination [6]. At the diabetes clinic of our hospital, the combination of metformin and glimepiride is commonly prescribed to manage T2D. However, both these drugs are known to influence autonomic functions [7, 8]. Metformin inhibits sympathetic activity by acting centrally on hypothalamic nuclei [7, 9, 10] and glimepiride promotes vagal tone, possibly stimulating the medullary cardioinhibitory center [8]. For the treatment of hypertension in diabetes, calcium channel blockers or angiotensin-converting enzyme (ACE) inhibitors are frequently prescribed as a single drug or in combination [11]. In our setup, amlodipine and enalapril are commonly used to manage hypertension in diabetes. Nonetheless, calcium channel blockers and ACE inhibitors are known to affect autonomic functions [12]. Calcium channel blockers inhibit central sympathetic outflow [13], whereas ACE inhibitors improve parasympathetic functions by acting centrally [14] and inhibit sympathetic activity mainly by reducing presynaptic vascular sympathetic outflow [15]. To date, there are no reports of assessment of autonomic functions or dysfunctions in T2D patients receiving these antidiabetic and antihypertensive drugs. It is not known why T2D patients develop CV dysfunctions within a few years of acquiring diabetes despite adequate treatment by these drugs. Moreover, the link of autonomic dysfunctions to cardiometabolic risk profile has not yet been assessed in T2D patients.

Baroreflex sensitivity (BRS) and heart rate variability (HRV) have recently been reported as sensitive markers of CV risks in various clinical disorders [16–19]. We have reported the importance of BRS and HRV analysis in assessing cardiometabolic risks in newly diagnosed prehypertension, hypertension, prediabetes, and diabetes [20–23]. The raised level of osteoprotegerin (OPG), a glycoprotein chiefly secreted from vascular smooth muscle cells and adipocytes, has been linked to atherosclerosis and CVD [24]. Our previous report depicts a positive association of OPG with decreased HRV in the Indian population with T2D [25]. However, it is unclear whether OPG is altered in T2D patients treated with oral antidiabetic and antihypertensive drugs. Also, the link of alteration in OPG to attenuation of BRS has not been reported in patients with T2D with and without HTN.

Although alteration in HRV and BRS has been reported in DM [26], the magnitude of alterations in these two parameters in patients with T2D with and without hypertension on medical treatment has not been assessed. Also, BRS as a marker of CV risk in diabetes has not been evaluated. Therefore, in the present study, we have used BRS and HRV measurements for CV risk assessment in patients diagnosed with T2D for not less than 2 years and patients receiving metformin and glimepiride as combination therapy. Further, we have analyzed the pattern of CV risks in these patients with no hypertension or with hypertension and receiving oral amlodipine and enalapril as antihypertensive treatment. As factors such as adiposity and glycemic status in patients with diabetes are known to influence autonomic functions, including HRV [26–28], in the present study, we have included patients matched for age, gender, ethnicity, body mass index (BMI), blood glucose and glycated hemoglobin. We hypothesized that factors promoting CV risk in patients with T2D on oral antidiabetic drugs are different when associated with hypertension.

## Materials and methods

### Study design

This was a cross-sectional analytical study conducted in Biochemistry, Physiology, and Medicine departments at Jawaharlal Institute of Postgraduate Medical Education and Research (JIPMER), Puducherry, India. This study was first reviewed and approved by an institutional review board (JSAC), followed by Institutional Ethics Committee (Human studies: JIP/IEC/2018/305) before the study was conducted. Written informed consent was obtained from all participants at the time of recruitment.

#### Study participants

**Fig. 1:**
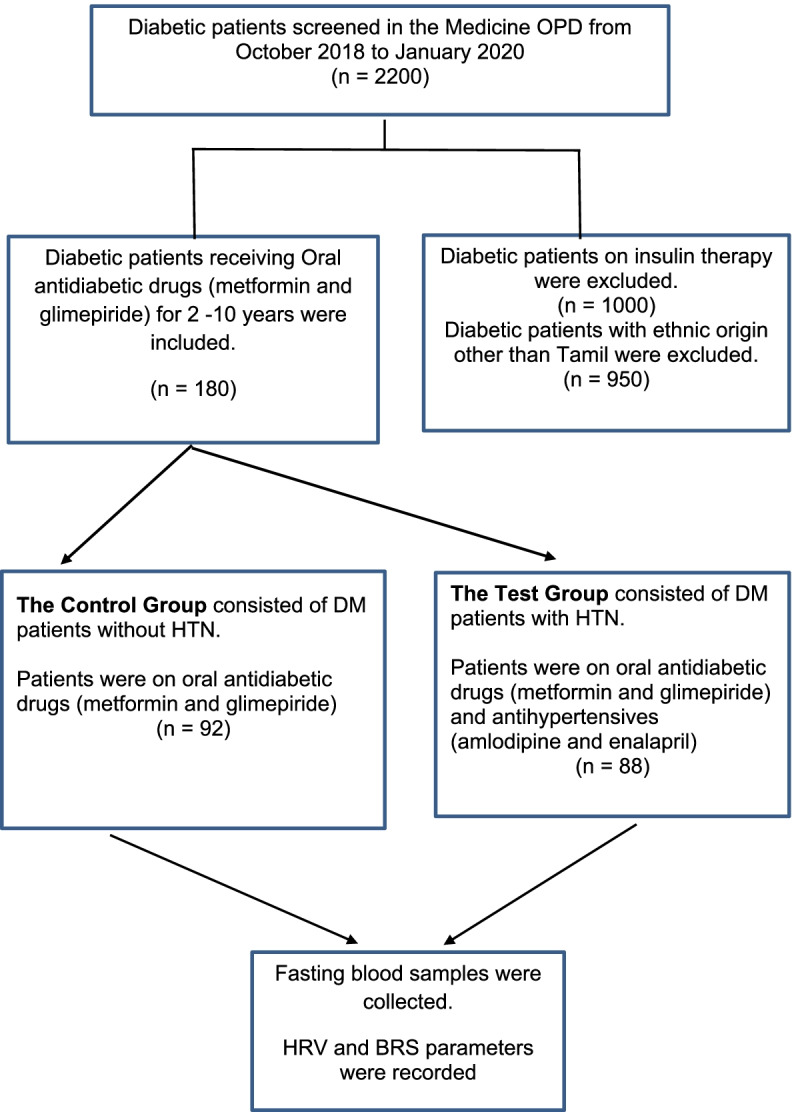
Flow chart of patients’ recruitment

A total of 180 T2D patients were recruited from the medicine out-patient department (OPD). We screened around 2200 diabetic patients between November 2018 and January 2020 and recruited 201 T2D patients following exclusion and inclusion criteria. Twenty-one patients with uncontrolled diabetes/hypertension were excluded from the study. Thus, the final sample size for the present study was 180. Among them, 92 patients had no hypertension and 88 patients had hypertension. Accordingly, they were divided into two groups: the control group (*n* = 92) consisting of T2D patients on treatment with oral antidiabetic drugs (OAD), and the test group consisting of T2D patients (*n* = 88) on treatment with antidiabetic and antihypertensive drugs (Fig. 1).

#### Inclusion criteria

To ensure uniform alteration in autonomic and metabolic functions by the antidiabetic drugs, the T2D patients receiving only the combination of metformin and glimepiride were contacted for the study. For the test group, T2D patients with history of hypertension who were on amlodipine and enalapril combination for hypertension were enrolled. Among the patients contacted, only those who were on antidiabetic and antihypertensive treatment for a period of two to 10 years and within the age range of 45 to 60 years, were recruited. All these patients had also received atorvastatin 5 or 10 mg daily.

#### Exclusion criteria

Patients with type 1 diabetes mellitus, patients on insulin therapy or glucocorticoid or immuno-suppressive therapy, BMI more than 35 Kg/m^2^, patients with history of CV disease, endocrine disorders, psychological, neurological disorders, or with features of acute infections were excluded. For the control group, T2D patients receiving OAD other than metformin and glimepiride were excluded during recruitment. The patients with uncontrolled diabetes and hypertension were also excluded from the study. The American College of Physicians guidelines of glycemic control of diabetic patients with pharmacological therapy was adopted for the purpose [29], and accordingly, those with HbA1c above 8% were excluded. The test group of T2D patients with hypertension receiving antihypertensive drugs other than amlodipine and enalapril combination were excluded. Also, those with systolic blood pressure above 160 mmHg and/or diastolic blood pressure above 100 mmHg were excluded.

### Sample size calculation

The sample size was calculated using the statistical formula for comparing means with equal variance. Considering the power as 80% to detect the difference (2.95) between two independent means of BRS (17.23 vs 9.07) based on the previous report [21] and a significance level of (alpha) at 0.05, the sample size calculated was minimum 88 in each group.

### Brief procedures

The study protocol was explained to the patients in their local language before obtaining written informed consent. All the participants were asked to avoid intake of coffee, nicotine, and alcohol 24 hours before the recordings. They were instructed to report next morning to the autonomic function test (AFT) lab between 8:30 am and 9:30 am in overnight fasting state. Fasting blood samples were collected the next day morning, and basal recordings were obtained 2 h after a light breakfast meal.

### Anthropometric measures

Basal demographic and anthropometric data were collected using a wall-mounted stadiometer to measure height, and a digital weight balance was used to measure the weight of individuals with light clothing. Body mass index was (BMI) derived using Quetelet’s formula. Basal demographic and anthropometric parameters of all the participants were recorded, and the personal history was noted using a structured data sheet.

### Recording of blood pressure

Patients reported in the autonomic function laboratory of Physiology Department at about 8.30 am. Their age, body weight, height, and BMI were recorded. Omron (SEM 1 Model), the automatic BP monitor apparatus (Omron Healthcare Co. Ltd., Kyoto, Japan), was used to measure their BP. The cuff size of the equipment used was 121 mm (width) × 446 mm (length), and the cuff tube length was 600 mm. The patients sat upright with backs straight on a wooden armed chair. After placing one forearm comfortably on the table, the middle of the arm of the subjects was coincided with the heart level. The BP cuff was tied on the arm roughly 2 cm above the cubital fossa, which was neither too tight nor loose. For each participant, heart rate (HR), systolic blood pressure (SBP), and diastolic blood pressure (DBP) were recorded at an interval of 5 minutes in each arm twice, and the mean of the four recordings was considered for each parameter.

### Recording of BPV parameters

The CV parameters and BRS were measured using the continuous BPV method (Finapres, Finometer version 1.22a, Finapres Medical Systems, Amsterdam, The Netherlands). Finapres is a non-invasive continuous hemodynamic CV monitor that uses the principle of finger plethysmography as described in detail in our earlier reports [18, 21]. The subjects were asked to lie down comfortably on a couch. The brachial cuff of Finapres was tied around the mid-arm about 2 cm above the cubital fossa, while the finger cuff of either medium or large size was tied around the middle phalanx of the middle finger, depending on the finger width. For their height correction, two sensors were placed, one at the heart level and another at the finger level. Following a 10 min supine rest, the continuous BPV recordings were obtained for the next 10 min [18, 21]. The reconstructed brachial pressure was acquired through a PC-based data acquisition system (Finapres Medical Systems BV, Amsterdam, The Netherlands). The parameters recorded from the reconstructed brachial pressure tachogram were HR, SBP, DBP, mean arterial pressure (MAP), rate-pressure product (RPP), inter-beat interval, left ventricular ejection time (LVET), stroke volume (SV), cardiac output (CO), total peripheral resistance (TPR) and BRS.

### HRV recording

For the recording of short-term HRV, the HRV measurement and analysis procedures described earlier were followed [22, 23]. ECG electrodes were connected to the four limbs. Lead II ECG was acquired at a rate of 1000 samples/s during supine rest using BIOPAC MP 100 data acquisition system (BIOPAC Inc., USA). The data were transferred from BIOPAC to the windows-based PC using AcqKnowledge software version 3.8.2. Ectopics and artifacts were eliminated from the recorded ECG. From the edited 256 seconds ECG recording, RR tachogram was extracted using the R wave detector in the software, and the data was saved in ASC-II format for later offline use for short-term HRV analysis. HRV analysis software (version 1.1., Biomedical signal analysis group, University of Kuopio, Finland) was used to analyze the frequency spectrum components. The frequency-domain indices of HRV included total power (TP), low-frequency power expressed in normalized units (LFnu), high-frequency power expressed in normalized units (HFnu), and the ratio of low-frequency to high-frequency power (LF-HF ratio); and the time-domain indices included mean standard deviation of RR intervals (SDNN), square root of the mean of the sum of the squares of the differences between adjacent RR interval (RMSSD), adjacent RR interval differing more than 50 milliseconds (NN50), and NN50 counts divided by all RR intervals (pNN50).

### Assessment of biochemical parameters

Following overnight fasting, five ml of venous blood was collected under aseptic precautions in clot activator tubes. Blood samples were centrifuged for 15 min after coagulation at 2500 RPM. Serum separated was processed for fasting glucose and lipid profile. Fasting serum glucose (FSG) was estimated by the Hexokinase method and lipid profile was measured by an automated AU5800 chemistry analyzer using commercial kits (Beckman Coulter AU5800, Beckman Coulter Inc., Brea, California, USA). LDL-C was analyzed by direct estimation. Total cholesterol, HDL-C, and triglyceride were analyzed by cholesterol oxidase, direct immunosuppression method, and Lipase/Glycerol phosphate oxidase method, respectively.

### ELISA parameters

TNF-α (Diaclone, France), insulin (Calbiotech, USA), and OPG (Fine test, China) were estimated by an enzyme-linked immunosorbent assay (ELISA). In the method of ELISA for OPG, the polyclonal goat anti-human OPG antibody binds to human endogenous OPG. Thus, the monomeric and dimeric OPG form a complex with the antibody. The washing step removes the nonspecific bound materials, while a second antibody complexed with streptavidin-HRP detects this complex, which follows the principle of sandwich ELISA. The concentration of OPG is directly determined by the standard dose-response curve. HOMA-IR was calculated using formula. HOMA-IR = Glucose X Insulin / 405 [30]. The inter-assay and intra-assay coefficients of variation for ELISA parameters were less than 3.5 and 6.1%, respectively.

### Statistical analysis

All the clinical characteristics and variables were expressed in frequencies and percentages for categorical variables. Continuous variables were expressed as Mean ± SD. The normality of data was checked by the Shapiro-Wilk test. Chi-square test was used for comparison between two groups for all the nominal data. For comparison between continuous parameters, Student’s unpaired t-test and Mann–Whitney-U test was used for normally distributed variables and non-normally distributed variables, respectively. The relationship between the variables in each group was evaluated by Spearman’s rank correlation analysis. For assessing the independent contribution of variables to BRS, multiple regression analysis was used. This was followed by stepwise regression analysis to elucidate the most important variable contributing to alteration in BRS. All statistical tests were performed using SPSS version 20.0. *P* value < 0.05 was considered significant.

## Results

A total of 180 diabetes mellitus patients were recruited from October 2018 to January 2020. The distribution of age, gender, and other histories are shown in Table 1. No significant differences were found between the groups in their baseline characteristics such as age, gender, ethnicity, duration of disease and BMI, histories of alcohol intake and smoking, and family history of diabetes, hypertension and CVD.

**Table 1:** Comparison of demographic, anthropometric indices, basal heart rate (BHR), blood pressure (BP), heart rate variability (HRV) and blood pressure variability (BPV) parameters between diabetes mellitus (DM) patients on treatment) with and without hypertension (HTN)

Variables	Control Group (DM without HTN) (*n* = 92)	Test Group (DM with HTN) (*n* = 88)	***P*** values
**Demographic & anthropometric parameters**			
Age (Years)	50.83 ± 6.17	52.28 ± 5.98	0.112
Gender (M/F) ψ	52/4	56/32	0.363
Alcohol intake: n (%) ψ	38(41.3%)	31(35.2%)	0.445
Smoking: n (%) ψ	14(15.2%)	11 (12.5%)	0.669
F/H diabetes: n (%) ψ	43 (46.7%)	48 (54.5%)	0.302
F/H hypertension: n (%) ψ	35 (38%)	42 (47.7%)	0.228
F/H CVD: n (%) ψ	16 (17.6%)	21 (23.9%)	0.357
Duration of disease (Years)	4.75 ± 1.98	5.19 ± 1.64	0.105
BMI (Kg/m^2^)	25.46 ± 5.07	26.51 ± 3.84	0.120
**BPV parameters**			
BHR (beats per min)	74.21 ± 10.78	78.79 ± 10.18	0.004
SBP (mmHg)	118.72 ± 9.11	127.59 ± 9.91	0.000
DBP (mmHg)	76.54 ± 7.83	81.28 ± 6.89	0.000
MAP (mmHg)	90.60 ± 7.52	96.71 ± 6.28	0.000
RPP (mmHg/min)	88.28 ± 15.82	100.45 ± 14.54	0.000
SV (mL)	81.91 ± 15.92	88.84 ± 24.34	0.024
LVET (ms)	308.02 ± 49.85	322.81 ± 31.90	0.019
CO (L/min)	5.95 ± 1.28	6.71 ± 1.74	0.001
TPR (mmHg.min/l)	0.92 ± 0.21	1.06 ± 0.35	0.002
BRS (ms/mmHg) #	13.90 ± 5.27	6.76 ± 4.58	0.000
**HRV parameters**			
TP (ms^2^) #	1012.90 ± 316.18	625.88 ± 229.84	0.000
LFnu	54.63 ± 18.30	63.68 ± 23.05	0.004
HFnu	46.19 ± 17.65	34.44 ± 12.19	0.000
LF/HF	1.30 ± 0.49	1.93 ± 0.62	0.003
SDNN (ms)	30.55 ± 10.86	20.56 ± 7.90	0.000
RMSSD (ms)	26.73 ± 11.63	17.26 ± 7.98	0.000
NN50	12.27 ± 6.75	8.32 ± 3.48	0.000
PNN50	7.78 ± 5.97	2.98 ± 2.16	0.000

ψ Data expressed as n (%) analyzed by Chi-square test. Rest of the values are expressed as Mean ± SD. Comparison between the groups was done by unpaired Student’s t-test for parametric data and by Mann–Whitney U test for non-parametric data (#). *P* Value < 0.05 was considered significant

*M/F* Male/Female; *F/H* Family history, *CVD* Cardiovascular diseases, *BMI* Body mass index, *BHR* Basal heart rate, *SBP* Systolic blood pressure, *DBP* Diastolic blood pressure, *MAP* Mean arterial Pressure, *RPP* Rate pressure product, *TP* Total power of HRV, *LFnu Normalized* Low-frequency power of HRV, *HFnu Normalized* High-frequency power of HRV, *SDNN* Standard deviation of normal to normal interval, *RMSSD* Square root of the mean of the sum of the squares of the differences between adjacent NN intervals, *NN50* The number of interval differences of successive NN intervals greater than 50 ms, *PNN50* The proportion derived by dividing NN50 by the total number of NN intervals, *SV* Stroke volume, *LVET* Left ventricular ejection time, *CO* Cardiac output, *TPR* Total peripheral resistance, *BRS* Baroreflex sensitivity

A significant increase was observed in the HR, SBP, DBP, MAP, and RPP in T2D patients with hypertension (Table 1).

Among the BPV parameters, SV, LVET, CO, and TPR were significantly increased, while BRS was decreased in the test group (Table 1).

Among the HRV indices TP, SDNN, RMSSD, NN50, and pNN50 were significantly less in T2D patients with hypertension (Table 1). LFnu was significantly higher, HFnu was considerably lower, and LF/HF ratio was significantly higher in T2D patients with hypertension (Table 1).

The serum concentration of glycemic parameters, lipid profile, and atherogenic indices among study participants are presented in Table 2. Total cholesterol (TC) was similar, but HDL-C was low (Table 2) in T2D patients with hypertension. Among the lipid risk factors, TC/HDL-C, LDL-C/HDL-C, TG/HDL-C and atherogenic index of plasma (AIP) were significantly higher in the test group (Table 2). TNF-α and OPG levels were higher in T2D patients with hypertension (Table 2).

**Table 2:** Comparison of glycemic parameters, lipid profile, lipid risk factors and other biochemical markers between diabetes mellitus (DM) patients on treatment) with and without hypertension (HTN)

Variables	Control Group (DM without HTN) (*n* = 92)	Test Group (DM with HTN) (*n* = 88)	***P*** values
**Glycaemic Parameters**			
FSG (mg/dL)	121.04 ± 34.65	126.62 ± 29.49	0. 247
HbA1c (g%)	6.92 ± 0.56	7,15 ± 0.60	0.475
Insulin (μU/mL)	19.59 ± 12.82	25.82 ± 11.09	0. 001
HOMA-IR	5.80 ± 4.09	8.26 ± 4.60	0. 000
**Lipid profile**			
TC (mg /dL)	152.28 ± 34.21	161.99 ± 41.27	0.093
HDL C (mg /dL)	37.96 ± 6.88	34.66 ± 4.43	0.000
LDL C (mg /dL)	87.45 ± 32.13	97.05 ± 37.02	0.070
TG (mg /dL)	141.26 ± 63.83	151.31 ± 73.61	0.338
VLDL C (mg /dL)	29.55 ± 14.76	30.26 ± 14.72	0.754
**Lipid risk factors**			
Non HDL-C (mg/dL)	117.01 ± 34.94	127.32 ± 39.83	0.072
TG/ HDL- C #	3,85 ± 1.95	4.41 ± 2.13	0.072
TC/ HDL-C #	4.06 ± 0.87	4.69 ± 1.17	0.000
LDL-C/ HDL-C #	2.32 ± 0.80	2.81 ± 1.08	0.001
AIP	0.53 ± 0.21	0.59 ± 0.20	0.042
**Other biochemical markers**			
TNF-α (pg/ml) #	19.85 ± 8.89	25.29 ± 13.73	0.004
Osteoprotegerin (pg/mL) #	223.08 ± 103.86	287.60 ± 121.36	0.001

The values are expressed as Mean ± SD for parametric data. Comparison between the groups was done by unpaired Student’s t-test for parametric data and by Mann–Whitney U test for non-parametric data (#). *P* Value < 0.05 was considered significant

*FSG* Fasting serum glucose, *HOMA-IR* Homeostatic model assessment of insulin resistance, *TC* Total cholesterol, *TG* Triglyceride, *HDL* High density lipoprotein, *LDL* Low density lipoprotein, *VLDL* Very low-density lipoprotein, *Non HDL-C* Non HDL cholesterol, *AIP* Atherogenic index of plasma = log_10_[TG/ HDL- C], *TNF- α* Tumor necrosis factor alpha, *OPG* Osteoprotegerin

Decreased BRS was significantly associated with rise in HR (r = − 0.277, *p* = 0.009) and RPP (*r* = − 0.334, *p* = 0.001) in the test group (Table 3). It was negatively associated with TG/HDL and AIP in the test group and positively associated with TP, SDNN, RMSSD, pNN50, and TNF-α in both the groups. Decreased BRS was linked to rise in OPG (*r* = − 0.417, *p* = 0.000) and LF/HF (*r* = − 0.331, *p* = 0.002) and TNF-α (*r* = − 0.595, *p* = 0.000) in test group (Table 3). The variables contributing to the decrease in BRS are shown in Tables 4 and 5 for T2D patients with and without hypertension, respectively. The most significant independent contributor to BRS is demonstrated by subsequent stepwise regression analysis in the second columns of Tables 4 and 5, respectively. For T2D patients with hypertension (Table 4) the TNF-α (β = − 0.297, *p* = 0.000) and for T2D patients without hypertension (Table 5) OPG had significant contribution to decreased BRS (β = − 0.344, *p* = 0.004) in the test group.

**Table 3:** Spearman correlation of BRS with various parameters in treated T2D patients with and without hypertension

Parameters	DM without HTN (*n* = 92)		(DM with HTN (*n* = 88)	
	***r***	***P***	***r***	***P***
BHR	−0.175	0.094	−0.277	0.009
SBP	0.075	0.478	−0.239	0.025
RPP	−0.191	0.067	−0.334	0.001
HOMA-IR	0.168	0.109	−0.018	0.875
TG/HDL	0.240	0.024	−0.284	0.009
AIP	0.240	0.024	−0.284	0.009
LF/HF	−0.198	0.064	−0.331	0.002
TP	0.209	0.045	0.298	0.005
SDNN	0.264	0.011	0.518	0.000
RMSSD	0.289	0.005	0.267	0.012
NN50	0.146	0.164	0.028	0.794
pNN50	0.285	0.006	0.228	0.033
OPG	−0.240	0.036	−0.417	0.000
TNF α	−0.238	0.034	−0.595	0.000

*RPP* Rate pressure product, *BHR* Basal heart rate, *SBP* Systolic blood pressure, *FBG* Fasting blood glucose, *TG* Triglyceride, *HDL* High density lipoprotein, *AIP* Atherogenic index of plasma, *SDNN* Standard deviation of normal-to-normal interval, *RMSSD* Square root of the mean squared differences of successive normal to normal intervals, *CO* Cardiac output, *OPG* Osteoprotegerin

^*^ *P* Value < 0.05 was considered significant

**Table 4:** Regression analysis to assess the independent association of BRS (as dependant variable) with biochemical markers (as independent variables) in test group consisting of T2D patients with hypertension (*n* = 88)

Independent variable	Standardized regression coefficient Beta	95% confidence interval		***P*** Value
		Upper limit	Lower limit	
Multiple regression				
AIP	−0.071	−6.944	3.662	0.539
TNF α	−0.297	−0.189	−0.015	0.022
OPG	−0.182	−0.018	−0.003	0.167

The *p* value < 0.05 was considered significant

*AIP* Atherogenic index of plasma, *TNF α* tumor necrosis factor alpha, *Model adjusted for OPG* Osteoprotegerin. *BRS* Baroreflex sensitivity

In the second line analysis was by stepwise regression where AIP and OPG are excluded in the regression model

**Table 5:** Multiple regression analysis to assess the independent association of BRS (as dependant variable) with biochemical markers (as independent variables) in test group consisting of T2D patients without hypertension (*n* = 92)

Independent variable	Standardized regression coefficient Beta	95% confidence interval		***P*** Value
		Upper limit	Lower limit	
AIP	0.187	−0.728	10.419	0.087
TNF α	−0.062	−0.177	0.103	0.597
OPG	−0.344	−0.030	−0.006	0.004

The *p* value < 0.05 was considered significant

*BRS* Baroreflex sensitivity, *RPP* Rate pressure product, *AIP* Atherogenic index of plasma, *TNF- α* Tumor necrosis factor alpha, *OPG* Osteoprotegerin

In the second line analysis was by stepwise regression where AIP and TNF α are excluded in the regression model

## Discussion

In the present study, BRS was grossly reduced in the test group (T2D patients with hypertension, receiving both oral antidiabetic and antihypertensive drugs) compared to the control group (T2D patients without hypertension, receiving only oral antidiabetic drugs) (Table 1). BRS was significantly correlated with HR, SBP, and RPP in the study group but not in the control group (Table 3). These findings suggest that BRS gradually decreases with rise in blood pressure in T2D patients. BRS is an important parameter of blood pressure variability. Decreased BRS is also a marker of CV morbidity and mortality, as it primarily reflects the impact of central autonomic modulation as well as the elastic properties of arteries [18, 21, 31]. Therefore, the study group patients in the present report are more prone to CV risks than the control group patients. BRS testing has a higher sensitivity and specificity than conventional laboratory autonomic function tests, including HRV [32]. Though BRS has been suggested as a marker of diabetic cardiac autonomic neuropathy [33, 34], there is a paucity of report of alteration in BRS during treatment of patients having T2D with hypertension.

In a previous report, though it has been reported that BRS declines with an increase in the duration of T2D in individuals diagnosed between 2 and 14 years before, the exact mechanism of decreased BRS was not described [34]. They hypothesized that the glycemic variations might reduce the BRS via oxidative stress or endothelial dysfunction, although these parameters were not assessed in this study. In the present study, TNF-α was significantly increased in the test group compared to control group, and the degree of correlation of TNF-α with BRS was significantly high in the test group (*r* = − 0.595, *P* = 0.000) compared to the control group (*r* = − 0.238, *P* = 0.034) (Table 3). Further, multiple regression analysis demonstrated the independent contribution of TNF-α to BRS in the test group (Table 4) but not in the control group (Table 5). These findings suggest that the decline in BRS in hypertensive diabetic subjects is closely linked to increase in TNF-α. Pro-inflammatory cytokines, including TNF-α have been implicated in the causation of CV complications in T2D patients [35, 36]. Inflammation has been reported as an established mechanism that links oxidative stress or endothelial dysfunction with insulin resistance [36]. Thus, from findings of the present study it appears that inflammation could be a major triggering factor in the causation of hypertension in diabetic patients, and TNF-α could be the marker of the CV risks in these hypertensive T2D patients. As such, inflammation has been strongly documented in the pathophysiology of hypertension, and TNF-α has been identified as an important pro-inflammatory marker in hypertension [37, 38]. In the present study, TNF-α had no independent contribution to BRS in normotensive diabetic patients (Table 5), depicting that inflammation may not be a major CV risk factor in T2D patients without hypertension.

In the control group, OPG had significant independent contribution to BRS (Table 5). OPG in diabetes has been reported to be involved in atherosclerosis and micro- and macro-vascular complications [39–41]. In postmenopausal women, circulating OPG levels were observed to be significantly associated with diabetes independent of CV risk factors [42]. AIP was close to the significant level of association with BRS in normotensive diabetic patients as demonstrated by multiple regression (Table 5). Thus, the atherogenic lipid profile might be a contributing factor to the decrease in BRS in normotensive diabetic patients receiving metformin and glimepiride. As these patients were also receiving atorvastatin, the process of atherosclerosis might have been slowed in them. Therefore, AIP could have been under check to some extent. In these patients with T2D, the OPG could be a marker of atherosclerosis and CV risks. But it appears from the observations in this study that the diabetic patients receiving oral antidiabetic drugs still have some degree of inflammation, and the inflammatory milieu in these patients might have stimulated the sympathetic discharge and reduced vagal activity. Inflammation and oxidative stress have been established to play significant roles in the progression of diabetes and hypertension [43, 44], and inflammation has been proposed to mediate the rise in BP in diabetic patients [45]. As such, anti-inflammatory therapy has been tried in treating diabetes, especially to prevent vascular complications [46]. In the present study, TNF-α was significantly high in the study group subjects and had profound contribution to decrease in BRS in them.

There was considerable degree of sympathovagal imbalance (increased LF-HF ratio, Table 1) in these subjects, despite them receiving the calcium channel blockers and ACE inhibitors for the treatment of hypertension that are known to decrease sympathetic discharge and improve autonomic balance [47, 48]. Further, TP of HRV and time-domain indices (SDNN, RMSSD, NN50, and pNN50) were significantly reduced in the study group in comparison to the control group. TP indicates overall cardiac vagal modulation, and decrease in TP is a marker of CV risk [49]. Further, reduction in time-domain indices of HRV reflects the decrease in vagal drive [49]. The hypertensive diabetic patients had more resting heart rate, BP and RPP (Table 1). Increased heart rate and RPP in hypertensive patients are established CV risks [50]. Thus, it is evident that despite antihypertensive treatment, hypertensive patients with T2D are at the risk of CV events, which could be due to the persistence of an inflammatory milieu.

We assessed the contribution of these potential cardiovascular biomarkers (AIP, OPG and TNF-α) in the entire study population (*n* = 180) and we found that OPG (β = − 0.349, *P* = 0.000) had more significant association with decrease in BRS compared to TNF-α (β = − 0.203, *P* = 0.017) (Table 6). This indicates that the level of OPG is a major contributor to CV risk in diabetes. Yet, hypertension in diabetes attenuates the link of OPG to reduction in BRS (Table 4). Recently we have reported the link of increased level of OPG with decreased cardio-vagal modulation in T2D patients treated with oral antidiabetic drugs [25]. The findings of the present study corroborate with our earlier report of OPG as a marker of CV risks in diabetes, and in addition these findings indicate that TNF-α is a potential surrogate biomarker of CV risk in hypertensive patients with T2D on oral antidiabetic drugs.

**Table 6:** Regression analysis to assess the independent association of BRS (as dependant variable) with biochemical markers (as independent variables) in all T2D patients with and without hypertension (*n* = 180)

Independent variable	Standardized regression coefficient Beta	95% confidence interval		***P*** Value
		Upper limit	Lower limit	
AIP	0.055	−2.799	6.125	0.463
TNF α	−0.203	−0.194	0.019	0.017
OPG	−0.349	−0.028	−0.010	0.000

The *p* value < 0.05 was considered significant

*BRS* Baroreflex sensitivity, *RPP* Rate pressure product, *AIP* Atherogenic index of plasma, *TNF- α* Tumor necrosis factor alpha, *OPG* Osteoprotegerin

Diabetes and hypertension share several pathophysiologic mechanisms such as inappropriate activation of the renin-angiotensin-aldosterone system (RAAS), oxidative stress, impaired insulin-mediated vascular function, inflammation, increased sympathetic activation, dysfunctional immune responses, and abnormal renal handling of sodium [2, 51]. Persistent low-grade inflammation and oxidative stress in the adipose tissue results in increased production of angiotensinogen and angiotensin II that leads to the elevation in BP [52, 53]. Therefore, ACE inhibitors are usually preferred antihypertensive drugs in diabetes with hypertension [11]. Increased adipose tissue mass (obesity) and increased visceral adiposity are the key factors behind the coexistence of both diabetes and hypertension [2], and obesity is known to influence HRV and BRS [54]. An increase in body weight, especially the rise in abdominal obesity, typically follows the rise in blood pressure [55]. Therefore, in the present study we had recruited BMI-matched patients in both groups.

The glycemic status (levels of serum glucose and glycated hemoglobin) is known to influence HRV [56]. Therefore, in this study we had patients in both control and test groups matched for glycemic parameters (fasting serum glucose and glycated hemoglobin). However, on further biochemical investigations we found that insulin and HOMA-IR were significantly higher in test group compared with the control group (Table 2). Insulin is known to have a dubious relationship with hypertension, as physiologically it promotes vasodilation [57]; but with development of insulin resistance and impairment of associated PI3-kinase signalling, this vasodilatory effect is lost [58]. Insulin can increase tubular reabsorption of sodium and promote sympathetic nerve activity [59], thereby increasing basal blood pressure levels. Therefore, relative circulating hyperinsulinemia or insulin resistance at the tissue level could be the reason for associated hypertension in patients with T2D. In the present study, HOMA-IR was not significantly correlated with BRS in both groups. Hence, it is unlikely that insulin resistance could be a significant contributor to decreased BRS in patients with T2D having hypertension.

Though the major limitation of the present study is that we did not take a pure nondiabetic control group to compare cardiometabolic risks of diabetic patients with nondiabetic subjects, based on our previous data of control subjects [20, 21] we can infer that the cardiometabolic risks in all diabetic subjects in the present study are considerably higher. We also found the CV risk to be relatively higher in T2D patients with hypertension than those without hypertension. The BRS as observed in earlier studies [6, 20], is above 20 ms/mmHg in healthy normotensive-nondiabetic subjects. In the present study, we found BRS to be less than 15 ms/mmHg in T2D patients without hypertension and less than 10 ms/mmHg in T2D patients with hypertension. As the present study is of cross-sectional nature, BRS can’t be proposed as a physiological marker for prediction of hypertension in diabetes. Nevertheless, these data indicate that decreased BRS could be a physiological marker of CV risk in T2D patients on oral antidiabetic drugs, OPG could be a major contributor to the decreased BRS in these patients, and TNF-α might be a potential determinant of decline in BRS in T2D patients with hypertension receiving oral antidiabetic and antihypertensive drugs. Thus, it is evident that despite antidiabetic treatment, diabetic patients develop hypertension, which could be due to persistence of chronic low-grade inflammation in these patients. Further, these patients are at higher CV risks compared to the patients not having hypertension. The level of diabetes and hypertension control, not whether patients are under specific drugs, is the real predictors of CV risks. Therefore, studies should be conducted to evaluate if anti-inflammatory therapy can be considered as part of diabetes management to prevent the development of hypertension and to reduce the CV risks in these patients. Also, future follow-up studies should assess if the quantum of decrease in BRS could be a surrogate marker for CV risk assessment in diabetic patients receiving oral antidiabetic drugs.

## Conclusion

Despite antidiabetic and antihypertensive treatment, patients with hypertension and diabetes had more cardiometabolic risks compared to normotensive patients with diabetes. Inflammation could be the inciting factor for rise in BP and decreased BRS in hypertensive diabetics. Decreased BRS could be the marker of CV risk in T2D patients.

## Data Availability

The datasets analysed in this study are available with the corresponding author, which can be obtained on reasonable request.

## Abbreviations

T2D: Type-2 diabetes mellitus
CVD: Cardiovascular diease
OPG: Osteoprotegerin
TNF- α: Tumor necrosis factor alpha
OAD: Oral ant-diabetic drugs
HRV: Heart rate variability
AIP: Atherogenic index of plasma
BRS: Baroreflex sensitivity
TP: Total power
LF: Low-frequency
HF: High-frequency power
LH/HF: Low-frequency to high-frequency ratio
RPP: Rate pressure product
IR: Insulin resistance
BHR: Basal heart rate
